# Ethanol extract and chromatographic fractions of *Tamarindus indica* stem bark inhibits Newcastle disease virus replication

**DOI:** 10.1080/13880209.2017.1331364

**Published:** 2017-05-24

**Authors:** Omobola O. Okoh, Grace E. Obiiyeke, Uchechukwu U. Nwodo, Anthony I. Okoh

**Affiliations:** a SAMRC Microbial Water Quality Monitoring Centre, University of Fort Hare, Alice, South Africa;; b Department of Chemistry, University of Fort Hare, Alice, South Africa;; c Department of Botany, Delta State University, Abraka, Delta State, Nigeria;; d Applied and Environmental Microbiology Research Group (AEMREG), Department of Biochemistry and Microbiology, University of Fort Hare, Alice, Eastern Cape, South Africa

**Keywords:** Haemagglutination, folklore medicine, antiviral, allantoic fluid, tamarind

## Abstract

**Context:** The plethora of ethnomedicinal applications of *Tamarindus indica* Linn. (Leguminosae), tamarind, includes treatment of human and livestock ailments; preparations are recognized antipyretics in fevers, laxatives and carminatives. African folklore has various applications of tamarind. However, in Nyasaland, domestic fowl are fed with preparations for prophylactic properties.

**Objectives:** The objective of this study is to evaluate the antiviral properties of *T. indica* extract.

**Materials and methods:***Tamarindus indica* stem bark was extracted through ethanol maceration over 24 h, and the crude extract was fractionated by gravity-propelled column chromatography. Newcastle disease virus (NDV) inhibitory activity of extract and fractions were evaluated *in vivo* using 10-d-old embryonated chicken egg (ECE) as the medium for virus cultivation and antivirus assay. About 240 ECE were grouped into eight (three controls and five experimental) and, 200 μL of the extract and fractions respectively inoculated into NDV pre-infected eggs and incubated at 37 °C. Allantoic fluid was harvested 5 d post-virus infection and assayed for haemagglutination (HA).

**Results:** Anti-NDV assessment showed 62.5 mg/mL of crude extract and fractions: TiA, TiC and TiD to yield a HA titre of 1:128 each, while TiB showed 1:64 HA titre. At 125 mg/mL, a titre of 1:16 was recorded against TiB and TiD and, 1:8 against TiA. Similarly, crude extract and TiC, each recorded 1:4 HA titre. However, the minimum concentrations of extract and fraction for virus inactivation were 0.24 mg/mL and 0.49 mg/mL, respectively.

**Conclusion:** The antiviral activity shown by *T. indica* portends novel antiviral drugs and, perhaps, as scaffold for new drugs.

## Introduction

Folk medicine overtly relies on herbs in the treatment of ailments of infectious and non-infectious nature and this practice dates into antiquity as early men mostly relied on herbs for primary health care (Abdalla et al. 2012; Paulia et al. 2016). Understandably so, herbs have been the major source of several biological active compounds which serve as drugs or scaffold for drugs in current times (Zandi et al. 2013; Sethi et al. 2017). Besides the extra advantage of potentiating multiple targets (broad spectrum) and target sites (multiple mechanism of action) with minor side-effects, low resistance due to selective pressure of infective agents has been adduced to herbal remedies (Nwodo et al. 2011a). The cost effectiveness of herbal remedies implies affordability and may be the reason why most rural dwellers of the third-world economies rely upon herbal remedies for primary health care (Nwodo et al. 2011a). Consequently, *Tamarindus indica* Linn. (Leguminosae), referred to as ‘Tamarind’, was evaluated for antiviral properties following cultural applications in Nyasaland known today as Malawi (Williamson 1995). Documented folklore application of *T. indica* in the treatment of various human ailments includes inflammations, sore throat and as a liniment for rheumatism. Additionally, poultice made from the pulp is used for the alleviation of sunstroke, poisoning from *Datura* spp. L. (Solanaceae) and alcoholic intoxication. In the Asian sub-continent, the fruit is prescribed to counteract the ill effects of overdoses of *Hydnocarpus anthelmintica* Pierre (Flacourtiaceae) given in leprosy. Restoration of sensation in cases of paralysis has also been ascribed to *T. indica* pulp. In addition, ointment made of tamarind pulp has been reported to be used in purging domestic animals of vermin (Morton 1987). Veterinary folkloric applications of *T. indica* abound, and an important instance is the mixing of fowl feed with soaked stem bark of *T. indica* for various reasons including prophylactics.

## Materials and methods

### Plant material

The plant was collected in the month of April 2010 from the woods of More, Sokoto South Local Government Area, Sokoto State, Nigeria. The plant was taxonomically identified by Mr. J.O. Ozioko, a voucher specimen (TiN/01), was deposited at the Herbarium of the Department of Botany, University of Nigeria, Nsukka. The fleshy part of the stem bark was used in the experiment.

### Extraction of plant material

The pulverized dried stem back (50 g) was extracted by cold maceration in 400 mL of absolute ethanol (BDH) for 24 h. The mixture was filtered through a Whatman No. 1 filter paper, and the filtrate was concentrated by the evaporation to dryness in a steady air current. Subsequently, extracts were exposed to UV rays for about 18 h for sterility and thereafter, extracts were stored in sterile containers at room temperature.

### Crude extract fractionation

Crude extract was fractionated using a glass column of an internal diameter of 8 cm and a length of 100 cm (Quickfit, England, UK). Column grade silica gel (120–200 mesh size) was wet-packed, such that 2/3 height of the column was attained, using benzene and ethyl acetate (6:4) solvent system pre-determined with thin layer chromatography (Analytical TLC on silica gel G600 0.25 mm thickness). Crude extract (20 g) was dissolved in absolute ethanol (10 mL) and, then made into paste by mixing with silica gel (5 g). The slurry was placed into the column and eluted continuously with the mobile phase (benzene/ethylacetate 6:4) under gravity. Aliquots of 20 mL were collected and monitored by bands formed and distance travelled on TLC. Fractions with similar mobility on TLC were pooled and the four total distinct fractions were designated as TiA, TiB, TiC and TiD. The solvent in the fractions was evaporated under steady air current at room temperature.

### Virus strain

Velogenic Newcastle disease virus strain (KVD-NDV) was obtained from Nigerian Veterinary Research Institute (NVRI) Vom, Plateau State, Nigeria. The freeze-dried virus was re-suspended and activated in accordance with the methods of Nwodo et al. (2011b).

### Virus cultivation and antivirus assay

Ten-day-old embryonated chicken eggs (five replicates) were inoculated with 200 μL of four hemagglutinating unit (HAU) test virus strains (≈ 1:128 virus titre) in accordance with the standards of the organization international des epizooties [OIE] (2004). Subsequently, 200 μL of 250 mg/mL of the crude ethanol extract of *T. indica* stem bark was inoculated and the eggs were incubated at 37 °C in a humidified incubator. The eggs were candled daily to observe embryo viability. Eggs with dead embryo within 24 h post-inoculation were discarded but the allantoic fluid of eggs with dead embryo 24 h post-inoculation were harvested following 4 h chilling at +4 °C and spot-checked for haemagglutination (HA). Eggs with viable embryo at 120 h were likewise chilled at +4 °C, the allantoic fluid was harvested and spot-checked for HA. Allantoic fluid harvests were pooled into sterile sample bottles, chilled at −20 °C and stored until further use. A total of 240 embryonated chicken eggs were used for the study. The eggs were grouped into eight thus, representing three controls, four fractions and the crude extract. Each group had about 30 eggs which were sub-grouped into six with each sub-group having five eggs in accordance with the treatment levels (extract and fraction concentration).

In addition to the *T. indica* antiviral assay, about 200 μL of three different concentrations (250, 125 and 62.50 mg/mL) of the crude extract and four chromatographic fractions (TiA, TiB, TiC and TiD) were inoculated into 10-d-old embryonated chicken egg. Similarly, the minimum concentrations of the *T. indica* extract and fractions with inti-NDV activity were quantitatively assessed. Virus and extract (crude and fractions) inoculations were through the allantoic and the chorioallantioc routes. The vents on the eggs were sealed with Wax-Vaseline mixture and re-incubated in a humidified incubator in the same condition as stated earlier. Allantoic fluid harvest and storage were as previously described.

All allantoic fluid harvests were tested for HA using the micro-titration method (OIE 2004). Two-fold dilutions, of 50 μL volumes of the virus suspension in allantoic fluid, were made with phosphate-buffered saline (PBS) as the diluent in 96-well U-shaped bottom microtitre plates (Sigma, St. Louis, MO) to obtain a range of 1:2–1:2048 dilutions. Then, 50 μL of 0.6% washed chicken red blood cells was added to all the wells. The last three wells served as the controls: virus control (10th well), extract control (11th well) and PBS control (12th well). The content of the wells was mixed by gently tapping the plates at the edges and then incubating at room temperature (∼27 °C) for 45 min after which the plates were examined for HA starting with the control using a plate reader (Flow Laboratories, Bradenton, FL). The end point was taken as the last dilution showing 100% HA; the titre was expressed as the reciprocal of the dilution and designated one hemagglutinating unit (HAU_100_).

## Results

The virus replication inhibition and/or virucidal activity of *T. indica*, stem bark, crude ethanol extract and column chromatographic fractions evaluated on velogenic NDV (KVD-NDV) showed positive activity. The allantoic fluid harvests treated with 62.50 mg/mL of the crude ethanol extract of the stem bark and fractions; TiA, TiB, TiC and TiD, each showed a positive spot HA while at a higher concentration of 125 mg/mL, only fractions TiA, TiB and TiD showed positive HA (Table 1). However, all velogenic NDV-infected eggs treated with 250 mg/mL concentration of both crude and fractions yielded allantioc fluids which showed negative spot HA.

**Table 1.** Allantioc fluid spot haemagglutination (HA) screening for antiviral activities of *T. indica* crude extract and fractions on velogenic NDV.

Concentration (mg/mL)	Crude	TiA	TiB	TiC	TiD	Virus control	Control (no virus, no extract)	Extract control
62.5	+	+	+	+	+	+	−	−
125	−	+	+	–	+	+	−	−
250	−	−	−	−	−	+	−	−

−: absence of HA activity.

+: presence of HA activity.

A quantitative assessment of the effects of crude and fractions showed that at concentrations of 62.5 mg/mL, the crude extract and fractions; TiA, TiC and TiD showed a titre of 1:128 each, while TiB showed neither increase nor decline in HA titre as the value remained 1:64 which was the titre used for the infection. At 125 mg/mL concentration, a titre of 1:16 was recorded against TiB and TiD. Also, at the same concentration, a titre of 1:8 recorded against TiA while the crude extract and TiC, each recorded 1:4 HA titre. Lastly, at 250 mg/mL, no HA was demonstrated in all the allantoic fluid harvests. However, the minimum concentrations for virus inactivation were 0.24 mg/mL for the crude extract, TiA and TiC and, 0.49 mg/mL was likewise recorded against TiB and TiD, respectively. The controls were groups with extract and fraction alone, virus alone and lastly, group with neither virus nor extracts (Table 2).

**Table 2.** Allantioc fluid haemagglutination (HA) titration assay for antiviral activities of *T. indica* crude extract and fractions on velogenic NDV.

Concentration (mg/mL)	Crude	TiA	TiB	TiC	TiD	Virus control	Control (no virus, no extract)	Extract control
0	1:256	1:256	1:256	1:256	1:256	1:256	0	0
62.5	1:128	1:128	1:64	1:128	1:128	1:256	0	0
125	1:4	1:8	1:16	1:4	1:16	1:256	0	0
250	0	0	0	0	0	1:256	0	0

0: absence of HA activity.

## Discussion

The concentration of 250 mg/mL both crude and fractions of the ethanol extract of the stem bark of *T. indica* showed complete inhibition of the hemagglutinating activity of velogenic NDV grown in chicken embryo. The preliminary investigation of the antiviral properties of *T. indica* following spot HA assay revealed that both crude and fraction TiC showed inhibition to the multiplication of NDV at a concentration of 125 mg/mL, and this could be interpreted to mean higher activity as compared with other fractions since similar results were not obtained. However, when the critical assay was carried out by titration of allantoic fluid harvest, antiviral activity was observed at all levels. The lowest concentration of extracts caused reduction in the replication of the virus; but the degree of inhibition varied with extract, fractions and sub-fractions. Fraction TiC and the crude extract showed similar activity, suggesting that the main component(s) with potent antiviral activity separated into fraction TiC. To buttress this point, when compared with other fractions TiC and the crude extract showed higher inhibition to the growth of NDV. Fraction TiA was next in terms of antiviral activity while other followed suit as was shown in the results. These observations are in consonance with the views of other works which have shown plant active chemicals including tannins, flavonoids and terpenes as possessing antiviral potentials (Edziri et al. 2012; Duan et al. 2014; Zhang et al. 2014). The specific chemical constituent of *T. indica* mediating antiviral activity was not established in this research nonetheless, it is a subject for future studies. Additionally, the minimum concentrations of the extract and fractions showing virus replication inhibition did not lead to the inhibition of hemagglutionation formation. However, further virus replication was observed to have been impaired with these concentrations as the virus titres did not increase over time in a viable embryo. As such, it may be inferred that the virus was inactivated but the hemagglutinin/nuraminidase structure was not disrupted.

## Conclusion

The anti-NDV activity demonstrated by *T. indica* indicates immense potentials towards harnessing active constituents of the plant for antiviral drugs. Although the antiviral constituent(s) of *T. indica* may have been active against hemagglutinin and/or neuraminidase thus, accounting for the absence of HA after *T. indica* treatment. Perhaps other components relevant for viral replication, including enzymes, were also impaired. However, this conclusion cannot be inferred with the findings of this work as further investigation is required. Nonetheless, it will be pertinent to isolate and characterize the active antiviral principles in *T. indica* as to ascertain novelty, and as well add to the existing body of knowledge.
